# Complexin Suppresses Spontaneous Exocytosis by Capturing the Membrane-Proximal Regions of VAMP2 and SNAP25

**DOI:** 10.1016/j.celrep.2020.107926

**Published:** 2020-07-21

**Authors:** Jörg Malsam, Simon Bärfuss, Thorsten Trimbuch, Fereshteh Zarebidaki, Andreas F.-P. Sonnen, Klemens Wild, Andrea Scheutzow, Lukas Rohland, Matthias P. Mayer, Irmgard Sinning, John A.G. Briggs, Christian Rosenmund, Thomas H. Söllner

**Affiliations:** 1 Heidelberg University Biochemistry Center (BZH), Im Neuenheimer Feld 328, 69120 Heidelberg, Germany; 2 Neuroscience Research Center, Charité Universitätsmedizin Berlin, Chariteplatz 1, 10117 Berlin, Germany; 3 European Molecular Biology Laboratory, Meyerhofstraße 1, 69117 Heidelberg, Germany; 4 MRC Laboratory of Molecular Biology, Francis Crick Avenue, Cambridge Biomedical Campus, Cambridge CB2 0QH, UK; 5 Center for Molecular Biology of Heidelberg University (ZMBH), Im Neuenheimer Feld 282, 69120 Heidelberg, Germany

## Abstract

The neuronal protein complexin contains multiple domains that exert clamping and facilitatory functions to tune spontaneous and action potential-triggered synaptic release. We address the clamping mechanism and show that the accessory helix of complexin arrests assembly of the soluble N-ethylmaleimide-sensitive factor attachment protein receptor (SNARE) complex that forms the core machinery of intracellular membrane fusion. In a reconstituted fusion assay, site-and stage-specific photo-cross-linking reveals that, prior to fusion, the complexin accessory helix laterally binds the membrane-proximal C-terminal ends of SNAP25 and VAMP2. Corresponding complexin interface mutants selectively increase spontaneous release of neuro-transmitters in living neurons, implying that the accessory helix suppresses final zippering/assembly of the SNARE four-helix bundle by restraining VAMP2 and SNAP25.

## Introduction

**Figure 1:**
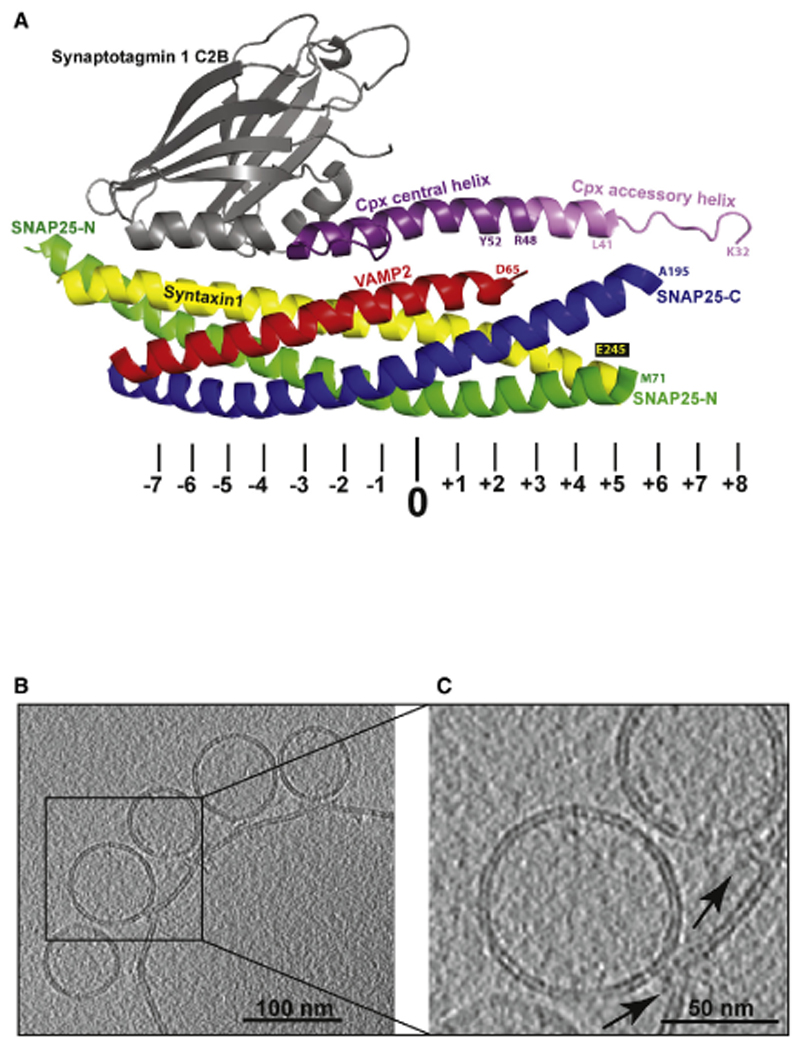
Cpx-Syt1 Primed SNAREpins and Docked Vesicles (A) Crystal structure of the membrane-distal Cpx-Syt1-SNARE interface (accession number PDB: 5W5C; modified from Zhou et al., 2017). The positions of the hydrophobic layers and the central ionic 0 layer within the SNARE motifs are indicated below. The second C2B binding site facing SNAP25 is not shown. (B) Slice through a cryo-tomogram of docked SUVs, whose fusion can be triggered by Ca^2+^. GUVs containing full-length t-SNARE complexes (syntaxin1/SNAP25) were mixed with SUVs containing Syt1 and the v-SNARE VAMP2 in the presence of WT CpxII. Samples were pre-incubated for 30 min on ice to efficiently accumulate the Cpx-stabilized prefusion intermediate. (C) Magnification of the area outlined in (B). Arrows indicate the position of putative SNAREpins and associated proteins.

Signal propagation between neurons relies on fast quantal release of neurotransmitters from the presynaptic terminal into the synaptic cleft. An incoming action potential elicits influx of Ca^2+^ into the nerve terminal, which instantly triggers fusion of neurotransmitter-filled synaptic vesicles docked at the active zone of the presynap-tic plasma membrane (Rothman, 2014; Südhof, 2013). The underlying core fusion machinery is formed by *trans* v/t-soluble N-ethylmaleimide-sensitive factor attachment protein receptor (SNARE) complexes (SNAREpins) bridging the vesicular and plasma membrane (Söllner et al., 1993; Weber et al., 1998). In a zipper-like manner, 16 layers of the α-helical SNARE motif assemble into a four-helix bundle, pulling the opposing membranes together to drive bilayer fusion (Figure 1A; Stein et al., 2009;Sutton etal., 1998).The vesicle (v)-SNARE VAMP2/synapto-brevin, anchored to synaptic vesicles, provides one helix, and the target (t)-SNARE (syntaxin1 and SNAP25), localized to the pre-synaptic plasma membrane, contributes three helices. SNAREpin formation is precisely controlled by regulatory proteins accelerating and arresting distinct assembly steps (Brunger et al., 2018; Jahn and Fasshauer, 2012; Südhof, 2013). Crucially, the Ca^2+^ sensor synaptotagmin1 (Syt1), which is anchored to synaptic vesicles,andthesmall cytosolic protein complexin Cpx)arrestSNAR-Epin assembly at a latemetastable state,a prerequisite for the hallmark fast Ca^2+^ synchronization (Fernández-Chacón et al., 2001; Geppert et al., 1994; McMahon et al., 1995; Melia, 2007; Mohrmann et al., 2015; Trimbuch and Rosenmund, 2016). In the absence of Syt1, spontaneous release events are increased, and evoked exocytosis is impaired (DiAntonio and Schwarz, 1994; Geppert et al., 1994; Littleton et al., 1994; Pang et al., 2006). Syt1 contains two cytosolic C2 domains (C2A and C2B) that bindCa^2+^, anionic phospholipids, andSNAREs, likely forming olig-omeric assemblies and restraining SNAREpins to arrest the prefu-sion stage in the absence of Ca^2+^ (Bello et al., 2018; Brose et al., 1992; Davletov and Südhof, 1993; de Wit et al., 2009; Li et al., 2019; Perin et al., 1990; Zhou et al., 2017). In the presence of Ca^2+^, the C2 domains deform the membrane, exerting force on the SNAREpins, and membrane fusion is triggered (Chapman and Davis, 1998; Fernandez et al., 2001; Fernández-Chaco´ n et al., 2001;Hui et al., 2009; Martens etal., 2007; Wang etal., 2016).

Like Syt1, complexin controls spontaneous and evoked exocytosis. To which degree complexin suppresses/stimulates spontaneous release events is controversial because knockout or knockdown manipulations yield diverse results and show neuron-specific differences (Yang et al., 2013). In addition, in vertebrates, such as *Mus musculus*, the stimulatory role seems to dominate, whereas in invertebrates, such as *Drosophila melanogaster* and *Caenorhabditis elegans*, the inhibitory functiondom-inates (Hobson et al., 2011; Huntwork and Littleton, 2007; Lin et al., 2013; Lopez-Murcia et al., 2019; Maximov et al., 2009; Pang et al., 2006; Reim et al., 2001; Strenzke et al., 2009; Wragg et al., 2017; Xue et al., 2007). Complexin consists of an N-terminal stimulatory region (amino acids [aa] 1–25) that activates Ca^2+^-triggered release, an inhibitory accessory helix (aa 26–47) that suppresses spontaneous release, a central SNARE-binding helix (aa 48–74), and a largely unstructured C-terminal region (aa 75–134) (Buhl et al., 2013; Cho et al., 2010, 2014; Gong et al., 2016; Iyer et al., 2013; Kaeser-Woo et al., 2012; Kümmel et al., 2011; Lai et al., 2014, 2016; Martin et al., 2011; McMahon et al., 1995; Seiler et al., 2009; Snead et al., 2014; Wragg et al., 2013, 2017; Xue et al., 2007, 2010; Yang et al., 2015). The C-terminal region contains a short amphipathic helix that binds high-curvature membranes and modulates species-specific inhibition (Gong et al., 2016; Seiler et al., 2009; Snead et al., 2014; Wragg et al., 2013, 2017; Zdanowicz et al., 2017). Interestingly, a recent study using magnetic tweezers and truncated SNARE proteins revealed that aa 1–31 of CpxI slow down zippering of the linker regions connecting the SNARE motifs to their transmembrane regions (Shon et al., 2018). Thus, the exact nature and molecular interactions of the membrane proximal, N-terminal 47 amino acids containing activating and suppression functions still remain unclear.

Crystal and NMR structures of SNARE-Syt1, SNARE-Cpx, and SNARE-Cpx-Syt1 complexes have provided critical information about intermolecular interactions (Bracher et al., 2002; Brewer et al., 2015; Chen et al., 2002; Zhou et al., 2015, 2017). However, many of these structures reflect the postfusion stage or are based on truncated proteins to mimic potential prefusion stages. A recent crystal structure of a truncated, partially assembled SNARE complex revealed a Syt1-Cpx-SNARE interface localized around the membrane-distal region of the SNAREpin (Zhou et al., 2017). A second Syt1 C2B domain simultaneously binds the other side of the SNAREpin at the central region. Both interactions are required for synchronized evoked exocytosis. In contrast, the organization of the membrane-proximal SNARE regions and, in particular, the biophysical interactions of the inhibitory accessory helix in the prefusion stage remain elusive and led to proposals of several models. These include electrostatic repulsion of negatively charged membranes, stabilization of the central helix by the accessory helix, and direct Cpx-SNARE interactions also linking SNARE complexes (Choi et al., 2016; Giraudo et al., 2009; Krishnakumar et al., 2015; Lu et al., 2010; Prinslow et al., 2017; Radoff et al., 2014; Trimbuch et al., 2014; Xue et al., 2010; Zdanowicz et al., 2017).

## Results

### Identification of Interaction Partners of the CpxII Accessory Helix by Site- and Stage-Specific Photo-Cross-linking

Because Ca^2+^ triggers membrane fusion on a sub-millisecond timescale, we reasoned that only a few molecular rearrangements may suffice to release *a priori* weak constraints, which the Cpx accessory helix imposes on its putative binding partners. To covalently trap the complexin-SNARE interactions in the metastable prefusion conformation, we used a cross-linking approach to probe the local environment of the accessory helix on a single-amino-acid level with a spatial resolution of about 3 Å in the pre-and postfusion states. Among the four mammalian complexin paralogs, we chose to analyze CpxII, which controls exocytosis in the brain and in other tissues (Reim et al., 2001; Yamada et al., 1999).Wetook advantage of a well-characterized reconstituted proteoliposome fusion assay that allows accumulation of prefusion Cpx-clamped SNAREpins and that, upon Ca^2+^ addition, triggers fusion, culminating in fully assembled postfusion *cis*-SNARE complexes (Bharat et al., 2014; Malsam et al., 2012). Each of the 22 amino acids of the CpxII accessory helix (aa 26–47) was exchanged for the photo-activatable, unnatural amino acid benzoyl-phenylalanine (BPA) (Young et al., 2010). In the absence of photo-activated cross-linking, like in a standard mutagenesis approach, the functional contribution of each amino acid can be tested. Upon photoactivation, interaction partners can be identified, and SNAREpin zippering may become irreversibly arrested. Prefusion SNAREpins were accumulated by incubating small VAMP2/Syt1-containing liposomes (small unilamellar vesicles [SUVs]) mimicking synaptic vesicles with large syntaxin1/SNAP25-containing liposomes (giant unilamellar vesicles [GUVs]) mimicking the pre-synaptic plasma membrane in the presence of wild-type (WT) CpxII or the respective single BPA mutations. Figure 1B shows a slice through a cryo-tomogram of this Cpx-stabilized prefusion intermediate accumulating docked v-SNARE/Syt1 SUVs on the surface of a t-SNARE GUV. Note the electron-dense regions between the two opposing membranes at high magnification (arrows), likely representing arrested SNAREpins and associated proteins (Figure 1C). At some docking sites, the GUV surface protrudes toward the SUV, suggestive of a constrained, readily releasable state (Bharat et al., 2014).

**Figure 2:**
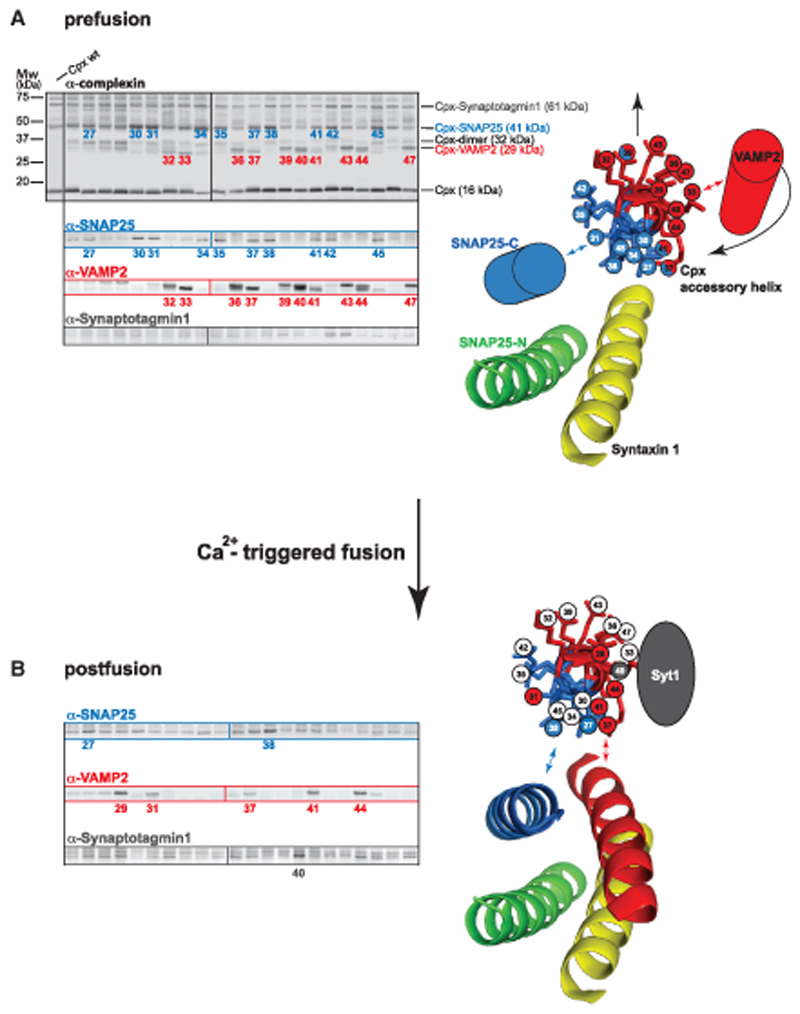
Mapping Interactions of the CpxII Accessory Helix with the Fusion machinery by Site- and Stage-Specific Cross-linking GUVs containing the full-length t-SNARE complex (syntaxin1/SNAP25) were mixed with SUVs containing Syt1 and the v-SNARE VAMP2 in the presence of WT CpxII or CpxII BPA mutants. Samples were preincubated for 30 min on ice to accumulate docked SUVs linked to the GUVs by Syt1 and *trans*-SNARE complexes. UV irradiation of the reaction mix was performed before (pre-fusion) and after triggering fusion with 100 μM calcium at 365 nm for 15 s on ice. Cross-link products were analyzed by western blotting using the indicated antibodies. Colored numbers indicate CpxII BPA mutants that show prominent cross-links to SNAP25 (blue), VAMP2 (red), and synaptotagmin (gray). Positions of identified cross-link products are indicated by the molecular identity. (A) Cross-link products identified at the prefusion stage (left panels). A view along the axis of the CpxII accessory helix shows interactions with SNAP25 (blue) and VAMP2 (red) (right panel). The membrane-proximal regions of VAMP2 and SNAP25 are depicted as cylinders because their prefusion structures are not known but may form α-helical structures. (B) Cross-link products identified at the postfusion stage and structural model.

To probe the local environment of the Cpx accessory helix within the prefusion SNAREpin, we subjected samples to UV (365 nm) irradiation, followed by western blotting and immunostaining. Detection of cross-linked products with an anti-Cpx antibody revealed specific cross-link bands at 29, 32, and 41 kDa and a weaker band at 61 kDa (Figure 2A). In addition, high-energy UV irradiation resulted in formation of spontaneous BPA-independent cross-links, as apparent in the sample containing WT Cpx, in particular at molecular weights above 50 kDa. The 32-kDa band indicates the presence of Cpx dimers formed by cross-links involving in particular the N terminus of the accessory helix, which may point toward the site of fusion pore formation. Specific products detected with the anti-Cpx antibody were co-recognized by anti-VAMP2, anti-SNAP25, and anti-Syt1 antibodies (Figure 2A), identifying inter-subunit cross-links. No prominent Cpx crosslink products could be detected using a syntaxin1-specific antibody (Figure S1A). Interestingly, formation of VAMP2- and SNAP25-Cpx cross-links follows a defined periodicity: pairs of two adjacent Cpx residues are linked to VAMP2 or SNAP25 in an alternating pattern, indicating localization of these SNAREs along two opposite sides of the Cpx accessory helix. Indeed, when viewed along the helix axis, the cross-links show that the Cpx accessory helix is sandwiched between SNAP25 and VAMP2 (Figure 2A). In summary, the data reveal the presence of a bipartite Cpx interface prior to fusion, which follows a helical pattern and captures the C termini of SNAP25 and VAMP2, likely preventing SNARE complex zippering.

If this pattern is specific for the prefusion stage, then it should change after fusion. Thus, the systematic cross-link approach was repeated with samples where liposome fusion had been triggered with 100 μMfree Ca^2+^ prior to UV treatment (Figure 2B). The Cpx cross-link pattern changes profoundly. After fusion, cross-link formation is restricted to a narrow region of the Cpx helical wheel that projects toward different VAMP2 and SNAP25 interfaces, which is consistent with the position of Cpx in the postfusion crystal structure (Figure 2B; Chen et al., 2002). No obvious cross-links to Syt1 were observed in the prefusion state. However, at the postfusion stage, a single prominent cross-linked product of 61 kDa is generated by the Cpx residue A40BPA, which was recognized by an anti-Syt1 and an anti-Cpx antibody (Figure 2B; Figure S1B). Because Cpx A40BPA displays a prominent cross-link with VAMP2 at the prefusion state (Figure 2A), this finding suggests that a fusion/Ca^2+^ dependent conformational rearrangement now allows Syt1 to interact with Cpx at its VAMP2 pre-fusion interface.

To narrow down the region of SNAP25 that cross-links to the Cpx accessory helix in the prefusion state, we repeated the above experiments using C-terminally truncated SNAP25 constructs terminating at layer +6 (SNAP25 aa 1–194) or at layer +8 (SNAP25 aa 1–200) of the second SNARE motif (Figure S2A). When using SNAP25 aa 1–194, we saw no SNAP25-positive Cpx cross-link products, localizing the Cpx-interacting region to the 12 C-terminal residues of SNAP25 (Figure S2B). To map the VAMP2-interacting region, we performed a proteolytic analysis of VAMP2 cross-linked to CpxII R37BPA (Figure S2C). UV-irradiated samples were subjected to site-specific proteolytic cleavage by botulinum toxin B (BoNTB) and D (BoNTD), cleaving VAMP2 at distinct sites (Figure S2D). The proteolytic pattern allocates the cross-linked site to the last three layers (+6, +7, and +8) of the VAMP2 SNARE motif (Figure S2E). Thus, the binding sites of the Cpx accessory helix can be confined to the membrane-proximal C-terminal ends of SNAP25 and VAMP2. Interestingly, single-molecule optical tweezer measurements revealed that, in isolated t-SNARE complexes, layers +5 to +8 are frayed and, thus, may become a natural target for Cpx accessory helix binding (Zhang et al., 2016). In turn, large parts of the Cpx accessory helix seem to be unstructured in the absence of the C-terminal SNARE regions (Figure 1A; Maximov et al., 2009; Trimbuch et al., 2014; Zhou et al., 2017). Therefore, the cross-linking data in combination with previous work suggest that the Cpx accessory helix and the membrane-proximal ends of SNAP25 and VAMP2 mutually influence each other’s structure, likely forming a short three-helix bundle that opposes the final zippering of the four-helix SNARE bundle (Figure 2). Such a model would overall be consistent with the observation that Cpx affects the SNARE assembly state and that an extended helical structure of Cpx is required for fusion arrest (Choi et al., 2016; Yin et al., 2016).

### Site-Specific Arrest of the Fusion Machinery by Cross-linking of the CpxII Accessory Helix Inhibits Ca^2+^ Triggered Membrane Fusion in a Reconstituted Assay

**Figure 3:**
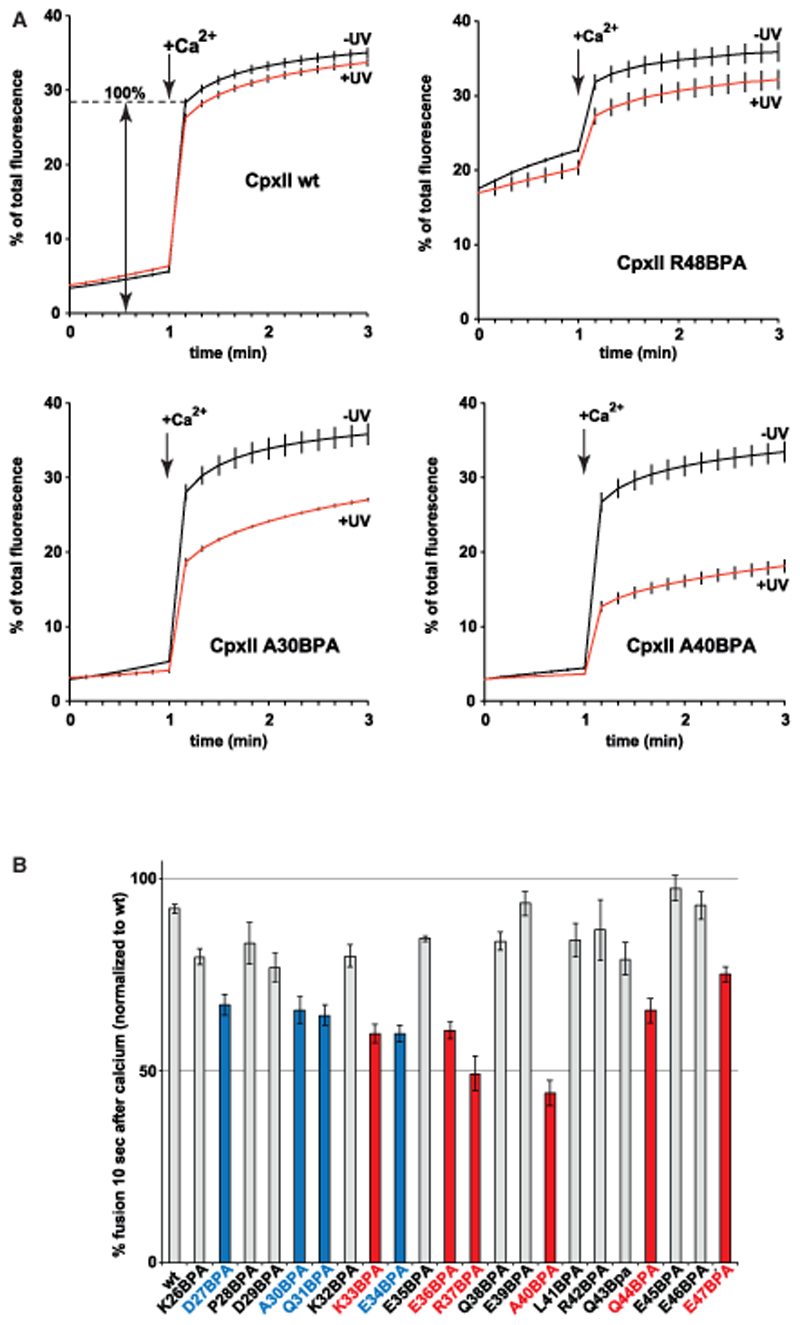
Site-Specific Arrest of the Fusion Machinery by Scanning BPA Cross-linking of the CpxII Accessory Helix Syntaxin1/SNAP25 GUVs were mixed with Syt/VAMP2 SUVs in the presence of WT CpxII or the indicated CpxII BPA mutants and incubated for 30 min on ice to dock vesicles. The reaction mixes were irradiated at 365 nm for 15 s on ice (control reactions without UV irradiation). Subsequently, fusion kinetics were recorded at 37°C for 1 min in the absence of Ca^2+^, and the measurement was continued for another 2 min after injection of 100 μM free Ca^2+^ to trigger fusion. (A) Lipid-mixing kinetics of WT CpxII and distinct CpxII BPA mutants with or without UV irradiation. CpxII R48BPA impairs SNARE complex binding, resulting in loss of the clamp and in an elevated starting signal. Error bars indicate SEM (n = 3). (B) BPA cross-link scan of the complete accessory helix and the effect on Ca^2+^-triggered fusion (signal change 10 s after Ca^2+^trigger as illustrated in A WT). Blue and red bar graphs indicate crosslinks to SNAP25 and VAMP2, respectively. Error bars indicate SEM (n = 3).

In functional terms, the site-specific cross-links should fix the complex in the prefusion state substantially interfering with subsequent Ca^2+^-triggered fusion events. We therefore compared untreated and UV-exposed samples in a lipid-mixing assay to monitor the effect of cross-link formation on liposome fusion kinetics. Irradiation of a control reaction containing WT CpxII confirmed that the nonspecific effects of UV exposure are negligible. In contrast, UV cross-linking of CpxII BPA mutants results in a variety of phenotypes showing moderate (A30BPA) or strong inhibition of fusion kinetics (A40BPA) (red curves in Figure 3A). Interestingly, in the absence of UV irradiation, replacement of single native amino acids by the bulky BPA at a few selective positions in the accessory helix reduced complexin’s clamping function but did not affect final Ca^2+^-triggered fusion (Figure S3). As a control, we introduced BPA at position R48, a residue in the central helix essential for overall binding of Cpx to the SNAREpin (Xue et al., 2007). This mutation resulted in loss of complexin function, abolishing clamp and Ca^2+^-dependent stimulation (Figure 3A).

We extended the analysis of UV-induced fusion arrest to all remaining residues of the CpxII accessory helix and normalized the data to themaximumfluorescence value of a control reaction containing WT CpxII, monitored 10 s after addition of calcium (Figure 3B). As expected, the pattern of UV-induced fusion inhibition correlated to a large extent with residues of the CpxII accessory helix that formed UV-dependent cross-links with SNAP25 or VAMP2, with maximum inhibition localized to the two adjacent residues R37 and A40 (compare Figures 3B and 2A).

To directly test whether Cpx-SNARE cross-link formation at the prefusion stage inhibits Ca^2+^-triggered SNARE complex assembly, we analyzed the ability of two CpxIIBPA mutants to prevent formation of SDS-resistant postfusion *cis* v/t-SNARE complexes (Hayashi et al., 1994). We compared CpxII A40BPA, a mutant in the accessory helix showing the strongest fusion inhibition with CpxII H52BPA, amutant localized to the central helix (Figure S4A). In the published postfusion structure, the corresponding residue Y52 in the CpxI isoform forms an interface with VAMP2 and Syntaxin1 (Chen et al., 2002). As expected, already at the prefusion state, CpxII H52BPA forms a cross-link product with Syntaxin1 (Figure S4B, lane 12). Importantly, the cross-link does not significantly affect Ca^2+^-triggered liposome fusion (Figure S4D), and only 14% of the CpxII H52BPA-Syntaxin1 cross-links are SDS sensitive in unboiled samples (Figures S4B, lane 13, and S4C). In other words, the CpxII H52BPA-Syntaxin1 cross-links do not prevent formation of SDS-resistant *cis* SNARE complexes. In contrast, 74% of CpxII A40BPA-VAMP2 cross-links remain sensitive to SDS (Figures S4B, lane 8, and S4C). Thus, the prefusion CpxII A40BPAVAMP2cross-links arrest *trans* SNAREpins and directly inhibit complete SNARE zippering upon addition of calcium, consistent with the observed fusion inhibition.

In addition to the biochemical assay, we analyzed the effect of cross-linking on Ca^2+^-triggered fusion in a morphological assay using cryoelectron microscopy (cryo-EM) and the CpxII A40BPA mutant. In the absence of Ca^2+^, approximately 10 SUVs were docked per GUV independent of cross-linking (Figures S5A and S5B). Ca^2+^ addition triggered efficient membrane fusion, and approximately one to two SUVs remained docked to the GUV surface. In contrast, UV irradiation profoundly abrogated Ca^2+^-triggered fusion of the BPA mutant, and approximately 10 SUVs remained docked per GUV. Thus, cross-linking efficiently arrests membrane fusion. Furthermore, it appears that our rather conservative evaluation of the biochemical data underestimates the inhibitory effects.

### Mutants Affecting the SNAP25/Cpx and VAMP2/Cpx Interface Selectively Increase Ca^2+^-Independent Fusion of Proteoliposomes and Spontaneous but Not Evoked Neurotransmitter Release in Neurons

**Figure 4:**
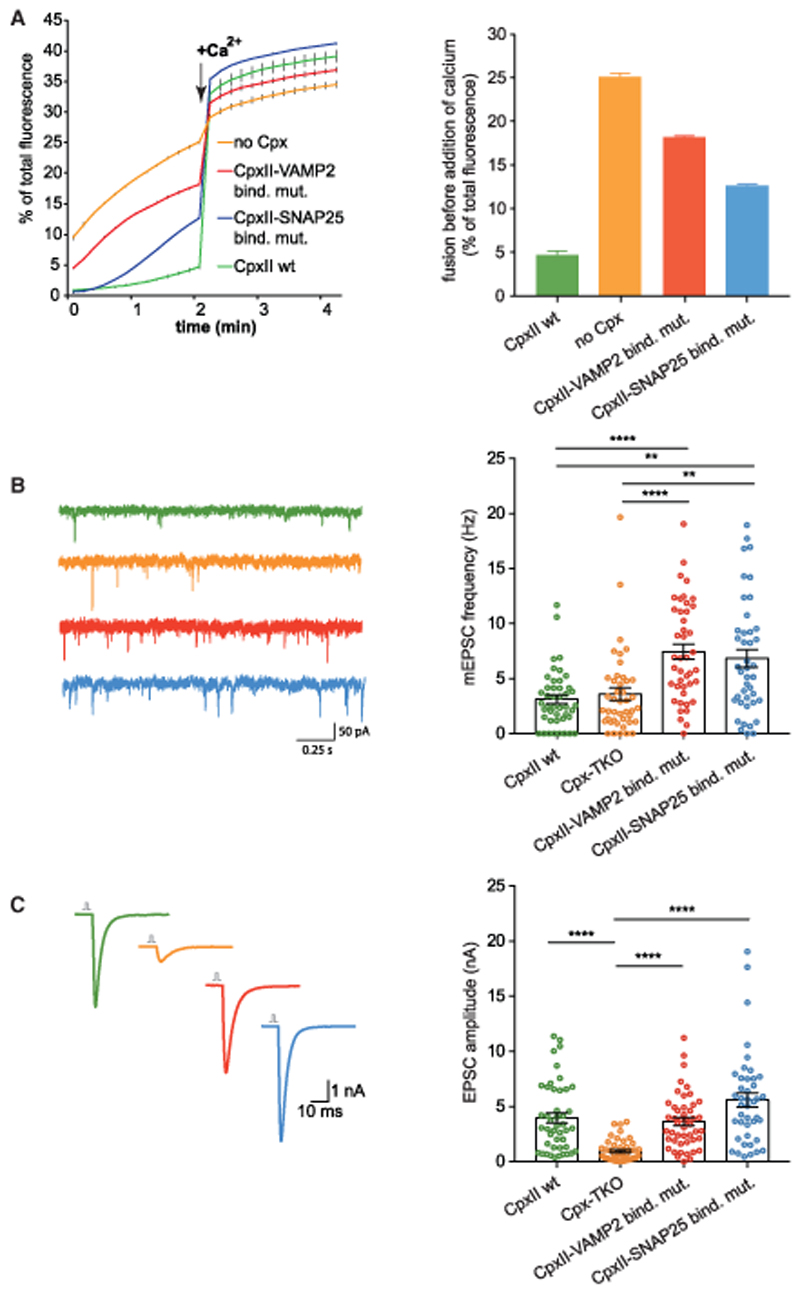
CpxII Quadruple Mutants of the SNAP25- and VAMP2-Binding Regions Selectively Increase Ca^2+^-Independent Fusion of Proteoliposomes and Spontaneous but Not Evoked Neurotransmitter Release in Neurons (A) t-SNARE-GUVs were mixed with Syt1/VAMP2 SUVs in the absence or presence of WT CpxII or the indicated mutants (CpxII-SNAP25-binding mutant: D27R, A30R, Q31E, E34R; CpxII-VAMP2 binding mutant: K33E, R37E, A40K, Q44E). Samples were pre-incubated for 5 min on ice, and then fusion kinetics were recorded at 37°C for 2 min in the absence of Ca^2+^. Fusion was triggered by injection of 100 μM free Ca^2+^. Error bars indicate SEM (n = 3). (B) Spontaneous release activity as determined by mean mEPSC frequency. n = 43 (Cpx TKO), n = 44 (WT CpxII), n = 43 (CpxII-VAMP2-binding mutant), n = 42 (CpxII-SNAP25-binding mutant) Error bars indicate SEM. **p < 0.01, ***p < 0.001, ****p < 0.0001. (C) Cpx TKO glutaminergic neurons were transduced with a lentivirus containing WT CpxII or the mutants. Shown is analysis of evoked responses by mean EPSC amplitudes. n = 47 (Cpx TKO), n = 45 (WT CpxII), n = 47 (CpxII-VAMP2-binding mutant), n = 42 (CpxII-SNAP25-binding mutant).

The question arises to which degree the interfaces identified by photo-cross-linking selectively affect fusion clamping. Therefore, we generated Cpx quadruple mutants containing reverse charge exchanges in SNAP25- or VAMP2-interacting residues. Circular dichroism analysis of the putative accessory central helix region (aa 21–83) showed a characteristic α-helical spectrum for both mutants. Although the spectra of WT Cpx and the VAMP2-binding mutant were almost identical, a reduced amount of molar ellipticity was detected for the SNAP25-binding mutant, indicating a decreased helical content of this mutant in the absence of its binding partners (Figure S6). Fusion reactions containing the SNAP25- or VAMP2-binding quadruple mutant revealed significant defects in suppressing calcium-independent fusion compared with WT Cpx (Figure 4A, initial 2 min). The increased vesicle consumption caused by unclamping apparently reduced the vesicle pool available for the Ca^2+^-triggered reaction (e.g., CpxII-VAMP2-binding mutant). Importantly, the reduced vesicle pools remained sensitive to the Ca^2+^ trigger, resulting in comparable fusion end signals, and the SNAP25 binding mutant even showed slightly increased fusion. This indicates that the stimulatory function of both complexin mutants in supporting Ca^2+^-triggered fusion by synaptotagmin is fully preserved. Altogether, the data reveal that these quadruple mutants primarily affect fusion clamping.

If the *in vitro* results are physiologically relevant, then, in neuronal exocytosis, the same Cpx mutants should predominantly affect spontaneous neurotransmitter release, leaving evoked release largely untouched. Therefore, we used lentiviral transduction of WT or mutant Cpx to perform rescue experiments on hippocampal glutamatergic neurons from Cplx 1/2/3 triple-knockout (TKO) mice (Trimbuch et al., 2014; Xue et al., 2007, 2008, 2010). Western blot analyses showed similar expression levels of all Cpx constructs (Figure S7A). As published previously, electrophysiological measurements using autapses confirmed that Cplx TKO profoundly reduced evoked neurotransmitter release, which could be rescued by WTCpx expression (Figure 4B). Both Cpx quadruple mutants rescued evoked release similar to WT Cpx. Neither Cpx mutants affected the readily releasable pool (RRP) or the release probability (Pvr) (Figures S7B and S7C). Short-term plasticity characteristics, analyzed by a train of action potentials at 50 Hz and 10 Hz, were rescued through expression of the Cpx mutants in the TKO. In line with rescue of Pvr, facilitation in the TKO was converted back to synaptic depression in all mutants tested (Figures S7E and S7F). In contrast to previous publications, the miniature excitatory postsynaptic current (mEPSC) frequency is unaffected by the Cplx TKO in this study, which can be explained by considerable variability between different synapses and cultures. Consistent with previous publications, loss of Cpx in hippocampal primary neurons does not increase the rate of spontaneous release (measured as the mEPSC frequency) because strong stimulatory functions of Cpx upon release can effectively counter the inhibitory functions (Figure 4C; Xue et al., 2008, 2009). In contrast, introduction of the VAMP2- or SNAP25-binding mutants into Cpx TKO neurons increased spontaneous release by a factor of two (Figure 4C). Thus, the interactions of the accessory helix with both SNARE interfaces specifically govern fusion clamping. Control experiments analyzing the amplitude of single spontaneous release events demonstrate that the Cpx mutants did not cause alterations in vesicle size or neurotransmitter loading, excluding indirect effects on spontaneous exocytosis (Figure S7D). Our data are consistent with electrophysiological analysis and molecular dynamics simulations analyzing Cpx synaptobrevin/VAMP2 interactions in *Drosophila melanogaster* (Bykhovskaia et al., 2013; Vasin et al., 2016).

## Discussion

Overall, our systematic, unbiased approach using site-specific photo-cross-linking revealed that, in the prefusion state, the accessory helix of CpxII forms a dual interface with the membrane-proximal regions of VAMP2 and the second SNARE motif of SNAP25. To identify these interactions, it was crucial to employ reconstituted full-length proteins in a proteoliposome fusion assay, maintaining the natural membrane constraints, which are characteristic of the prefusion site and also confine the structural organization of the fusion machinery. Although BPA exchange of complexin natural amino acids allowed us to identify the local environment of the accessory helix in the SNARE bundle, this approach can also be considered as a mutagenesis screen. In particular, non-conservative amino acid exchanges of key residues (e.g., R48 in Cpx) can perturb protein function. We detect and make use of such perturbations (e.g., R48BPA) in our functional assay in the absence of photoactivation. BPA substitutions at weak interaction sites or atamino acids flanking the actual binding site, however, are valuable tools to map protein interfaces, ideally at single-amino-acid resolution. In particular, the stage-specific changes in the cross-linking pattern and the close fit of the postfusion pattern with the known crystal structures confirm the value of the combined mutagenesis-cross-link approach. Thus, together with our functional studies *in vitro* and in living neurons, the data strongly suggest that both accessory helix interfaces are required for the clamping reaction.

Although there is general agreement that complexins stimulate evoked exocytosis, the physiological relevance of suppression of spontaneous fusion in mouse neurons is still debated. The techniques employed, such as neuronal cultures (autapses or interneuronal networks), and the complexin inactivation procedure (knockout or knockdown) may affect the outcome of the measurements (Maximov et al., 2009; Yang et al., 2013). In a recent publication, this issue was re-investigated using acute complexin I depletion in neuronal cultures derived from Cplx2/3 double-knockout mice, which are viable and fertile (Lopez-Murcia et al., 2019; Xue et al., 2008). This conditional depletion largely avoids indirect effects, such as collateral perturbations, compensatory processes, or aberrant synaptogenesis. Lopez-Murcia et al. (2019) showed that complexin knockout reduces spontaneous and evoked release in mouse hippocampal neurons, consistent with our electrophysiological studies using neurons derived from Cplx1/2/3 TKO mice.

Strikingly, our rescue experiments using Cpx mutants impaired in binding VAMP2 and SNAP25 reveal that, in addition to this stimulatory function of Cpx, the accessory helix mediates a prominent, suppressive clamping function. For spontaneous release to occur at WT frequency, synaptic vesicles need to enter a fully primed state, and this depends on the activating role of complexins. Our results suggest that the accessory helix acts as a built-in antagonist to restrict this strong activating function, establishing a decisive metastable state. The accessory helices of the membrane-anchored CpxIII and IV isoforms share only limited sequence homology with those ofCpxI and II. Nevertheless, these isoforms suppress spontaneous release events in bipolar cells of the retina (Mortensen et al., 2016; Vaithianathan et al., 2013). In these neurons, sequence adaptations of CpxIII/IV or other mechanisms may allow specific arrest of assembly of an alternative SNARE complex containing Syntaxin 3 (Morgans et al., 1996; Reim et al., 2005). Interestingly, direct SNARE interactions of the accessory helix seem not to be required in invertebrates such as *C*. *elegans*, where replacement of the accessory helix with a non-native helical sequence is sufficient to rescue the inhibitory function (Radoff et al., 2014). The phenotype of the VAMP2-binding quadruple CpxII mutant strongly suggests that, in mammalian neurons, evolutionary adaptations at the Cpx-SNARE interface may have been required to further stabilize the RRP.

Additional inhibitory functions of Cpx have been localized to its C-terminal region in invertebrates and vertebrates (Dhara et al., 2014; Kaeser-Woo et al., 2012; Makke et al., 2018; Wragg et al., 2017; Xue et al., 2009). Whether the C-terminal region and the accessory helix function as independent entities or directly affect each other remains to be shown. Because the stimulatory N-terminal region and the inhibitory C-terminal region interact with highly curved membranes, they may come into close proximity andmutually regulate their activities. The formation ofCpx dimers, as observed by our cross-linking study, and the observation that the Cpx unstructured regions fold backmake local cooperative effects plausible (Bowen et al., 2005; Gong et al., 2016; Snead et al., 2014; Zdanowicz et al., 2017).

**Figure 5:**
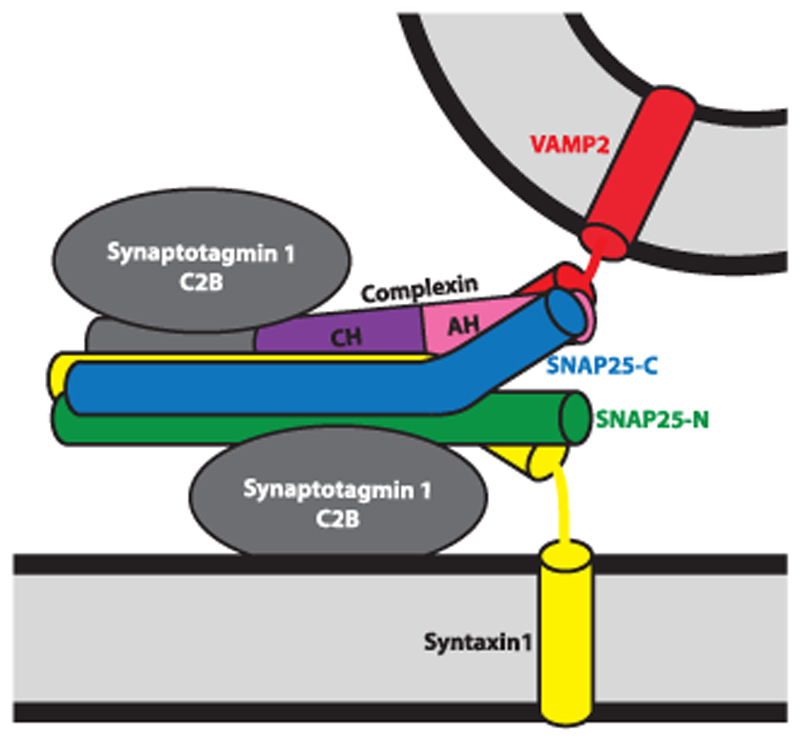
Model of How the Cpx Accessory Helix Clamps Fusion at the Synapse The central helix (CH) of Cpx stabilizes a partially zippered SNARE complex, and the accessory helix (AH) binds the membrane-proximal C-terminal ends of SNAP25 and VAMP2, preventing further SNARE complex zippering/assembly/membrane fusion. SNAP25-N and SNAP25-C indicate the first and second SNARE motif, respectively.

Overall, our study suggests a simple model of the accessory helix in which the membrane-proximal ends of VAMP2 and SNAP25 can form two alternative complexes: (1) a three-helix bundle with the Cpx accessory helix (Figure 5) or (2) a four-helix bundle with its syntaxin1 and SNAP25 partners allowing further SNARE zippering and initiating membrane fusion. The dual and apparently weak accessory helix-SNARE interactions have important implications: (1) by avidity, a sufficiently strong clamp is achieved; (2) the weak interactions will likely allow fast release upon arrival of the trigger; and (3) the three interaction partners may influence each other’s structure, generating a reaction intermediate, which may accelerate final SNAREpin zippering when unleashed by the Ca^2+^-Syt1 trigger. The Cpx accessory helix/VAMP2/SNAP25 interaction may also help to position the N terminus of Cpx relative to the membrane and SNAREpin, controlling its stimulatory role. How the Ca^2+^ influx triggers the structural transition remains to be determined. Direct Cpx-Syt1 interactions, as revealed by the cross-link in position 40 of CpxII, may play a role. Whatever the exact release mechanism may be, the identified Cpx-SNARE interactions make profound contributions to clamp spontaneous neurotransmitter release in the central nervous system, likely setting the correct threshold for neurotransmission and proper signal transduction in the brain.

## Star⋆Methods

### Key Resources Table

**Table:** 

REAGENT or RESOURCE	SOURCE	IDENTIFIER
Antibodies		
Rabbit polyclonal anti-Cplx1,2	Synaptic Systems	Cat# 122002; RRID: AB_122002
Rabbit polyclonal anti-Cplx1,2 SM195	this manuscript	N/A
Mouse monoclonal anti-β-Tubulin III	Sigma-Aldrich	Cat# T8660; RRID: AB_477590
Mouse monoclonal anti-Strep-tag	QIAGEN	Cat# 34850; RRID: AB_2810987
Mouse monoclonal anti-polyHistidine	Sigma-Aldrich	Cat# 1029; RRID: AB_260015
anti-VAMP2 rabbit polyclonal antibody 134	this manuscript	N/A
anti-syntaxin1 mouse monoclonal antibody HPC-1	Abcam	Cat# ab3265; RRID: AB_303654
horseradish peroxidase-conjugated goat anti rabbit IgG	Jackson ImmunoResearch Laboratories	Cat# 111-035-003; RRID: AB_2313567
horseradish peroxidase-conjugated goat anti mouse IgG	Jackson ImmunoResearch Laboratories	Cat# 115-035-146; RRID: AB_2307392
Alexa Fluor 680 goat anti-rabbit	Thermo Fisher Scientific	Cat# A21109; RRID: AB_2535758
IRDye 800CW Goat anti-mouse	LI-COR Biosciences	Cat# 926-32210; RRID: AB_621842
Bacterial and Virus Strains		
*E.coli* BL21(DE3)	Thermo Scientific	Cat# EC0114
pCMVdR8.91	Addgene	Cat# 2221
pVSV.G	Addgene #14888	Cat# 14888; RRID: Addgene 14888
f(syn)NLS-GFP-P2A-CpxII-WPRE	this manuscript	N/A
Chemicals, Peptides, and Recombinant Proteins		
4-Benzoyl-L-phenylalanine	Bachem	Cat# 4017649
Atto488 1,2-dipalmitoyl-sn-glycero-3-phosphoethanolamine	ATTO-TEC	Cat# AD 488-151
Atto550 1,2-dipalmitoyl-sn-glycero-3-phosphoethanolamine	ATTO-TEC	Cat# AD 550-151
1,2-dioleoyl-sn-glycero-3-phosphoserine	Avanti Polar Lipids	Cat# 840035P-10mg
1-palmitoyl-2-oleoyl-sn-glycero-3-phosphocholine	Avanti Polar Lipids	Cat# 850457P-25mg
1-palmitoyl-2-oleoyl-sn-glycero-3-phosphoethanolamine	Avanti Polar Lipids	Cat# 850757P-25mg
L-α-phosphatidylinositol	Avanti Polar Lipids	Cat# 840042P-25mg
L-α-phosphatidylinositol-4,5-bisphosphate	Avanti Polar Lipids	Cat# 840046P-1mg
cholesterol (from ovine wool)	Avanti Polar Lipids	Cat# 700000P-100mg
Nycodenz	PROGEN	Cat# 1002424
*n*-Dodecyl-β-D-maltosid	PanReac AppliChem	Cat# A0819
*n*-Octyl-β-D-glucopyranosid	neoLab	Cat# 1388GR500
Experimental Models: Cell Lines		
HEK293T	DSMZ	Cat# ACC 635
Experimental Models: Organisms/Strains		
Complexin 1,2,3 triple KO mice	Xue et al., 2008	N/A
Recombinant DNA		
pSK151	this manuscript	N/A
pSK130 (CpxII VAMP2 binding mutant)	this manuscript	N/A
pSK133 (CpxII SNAP25 binding mutant)	this manuscript	N/A
pJM112 (CpxII residue 21-83_wt)	this manuscript	N/A
pJM113 (CpxII residue 21-83_VAMP2 binding mutant)	this manuscript	N/A
pJM114 (CpxII_21-83_ SNAP25 binding mutant)	this manuscript	N/A
pTB16 (CpxII K26amber)	this manuscript	N/A
pTB17 (CpxII D27amber)	this manuscript	N/A
pTB18 (CpxII P28amber)	this manuscript	N/A
pTB19 (CpxII D29amber)	this manuscript	N/A
pJM48 (CpxII A30amber)	this manuscript	N/A
pTB20 (CpxII Q31amber)	this manuscript	N/A
pTB21 (CpxII K32amber)	this manuscript	N/A
pTB22 (CpxII K33amber)	this manuscript	N/A
pJM52 (CpxII E34amber)	this manuscript	N/A
pTB23 (CpxII E35amber)	this manuscript	N/A
pTB24 (CpxII E36amber)	this manuscript	N/A
pJM53 (CpxII R37amber)	this manuscript	N/A
pTB25 (CpxII Q38amber)	this manuscript	N/A
pTB26 (CpxII E39amber)	this manuscript	N/A
pJM49 (CpxII A40amber)	this manuscript	N/A
pJM54 (CpxII L41amber)	this manuscript	N/A
pTB27 (CpxII R42amber)	this manuscript	N/A
pTB28 (CpxII Q43amber)	this manuscript	N/A
pTB29 (CpxII Q44amber)	this manuscript	N/A
pTB30 (CpxII E45amber)	this manuscript	N/A
pTB31 (CpxII E46amber)	this manuscript	N/A
pTB32 (CpxII E47amber)	this manuscript	N/A
pTB40 (CpxII R48amber)	this manuscript	N/A
pSK41 (CpxII H52amber)	this manuscript	N/A
Software and Algorithms		
Axograph X	AxoGraph Scientific	RRID:SCR_014284
Prism 7	GraphPad Software	RRID:SCR_002798
Microsoft Office Excel 2010	Microsoft	RRID:SCR_016137
Image Studio Lite	LI-COR	RRID:SCR_013715
ImageJ 1.43u	National Institute of Health	RRID:SCR_003070
Ca-EGTA Calculator v1.3	MAXCHELATOR	N/A
Other		
PTFE O-ring (18 × 2 mm)	Dichtelemente arcus GmbH	Cat# 16536
Platinum-coated borosilicate glass wafer (40 mm × 27 mm)	GeSIM mbH	on request
Thermo Scientific Nunc F96 MicroWell Polystyrolplatte, weiß	Thermo Scientific	Cat# 236108
13 mm diameter conical tube adaptor for SW 55 Ti rotor	Eeckman	Cat# 358153

### Resource Availability

#### Lead Contact

Further information and requests for resources and reagents should be directed to and will be fulfilled by the Lead Contact, Thomas Söllner (thomas.soellner@bzh.uni-heidelberg.de).

#### Materials Availability

All unique/stable reagents generated in this study are available from the Lead Contact without restriction.

#### Data and Code Availability

This study did not generate any unique datasets or code.

### Experimental Model and Subject Details

#### Mice and preparation of cultured hippocampal neurons

All mouse experiments were performed in accordance with the regulation of the animal welfare committee of Charité-Universität-medizin Berlin and the Landesamt für Gesundheit und Soziales Berlin under license number T0220/09. Triple Cplx KO mice, in which all three genes were conventionally deleted (Xue et al., 2008), were used to prepare primary hippocampal neurons. As the full KO mouse line is not viable, mice were bred that were heterozygous KO for Cplx1 and homozygous KO for Cplx2 and Cplx3. Time pregnant females were sacrificed to obtain embryonic mice day 18 embryos. After genotyping, hippocampal neurons from triple KO neurons were prepared as described (Xue et al., 2007). Briefly, hippocampi were dissected and enzymatically treated using 25 units per ml of papain for 45 min at 37°C, followed by mechanically trituration to dissociate single neurons. For autaptic cultures, neurons were seeded on micro-island astrocyte feeder layers at low density (300 neurons cm^-2^) and for protein extraction on continental astrocyte feeder layers as high-density cultures (10.000 neurons cm^-2^). Astrocyte feeder layers were prepared 1-2 weeks before neuronal seeding, as described previously (Xue et al., 2007). Neuronal cultures were incubated in Neurobasal-A media supplemented with 50 μg/ml streptomycin and 50 IU/ml penicillin at 37°C.

### Method Details

#### Constructs

Full-length t-SNARE complex (syntaxin 1A 1-288, His_6_-SNAP25 1-206): The bicistronic expression plasmid (pTW34) encoding untagged full-length rat syntaxin 1A (1-288) and N-terminally His_6_-tagged mouse SNAP25 (1-206) was described previously (Parlati et al., 1999). The N-terminal extension including the His_6_-tag is highlighted in red.

syntaxin1A 1-288 sequence:

MKDRTQELRTAKDSDDDDDVTVTVDRDRFMDEFFEQVEEIRGFIDKIAENVEEVKRKHSAILASPNPDEKTKEELEELMSDIKKTANKVRSKLKSIEQSIEQEEGLNRSSADLRIRKTQHSTLSRKFVEVMSEYNATQSDYRERCKGRIQRQLEITGRTTTSEELEDMLESGNPAIFASGIIMDSSISKQALSEIETRHSEIIKLENSIRELHDMFMDMAMLVESQGEMIDRIEYNVEHAVDYVERAVSDIKKAVKYQSKARRKKIMIIICCVILGIIIASTIGGIFG

His_6_-SNAP25B 1-206 sequence:

MRGSHHHHHHGSMAEDADMRNELEEMQRRADQLADESLESTRRMLQLVEESKDAGIRTLVMLDEQGEQLERIEEGMDQINKDMKEAEKNLTDLGKFCGLCVCPCNKLKSSDAYKKAWGNNQDGVVASQPARVVDEREQMAISGGFIRRVTNDARENEMDENLEQVSGIIGNLRHMALDMGNEIDTQNRQIDRIMEKADSNKTRIDEANQRATKMLGSG

t-SNARE complex with C-terminally truncated SNAP25 (1-200/1-194): pTW34 (Parlati et al., 1999) was used as template DNA to truncate the SNARE motif at layer +6 (1-194) or around layer +8 (1-200) by introducing stop codons at the corresponding positions. 
   position: 1              171               194    200   206
   layer:                   −1 0  +1 +2 +3 +4  +5  +6 +7 +8
1-206:      MAEDAD........IDTQNRQIDRIMEKADSNKTRIDEANQRATKMLGSG
1-200:      MAEDAD........IDTQNRQIDRIMEKADSNKTRIDEANQRAT
1-194:      MAEDAD........IDTQNRQIDRIMEKADSNKTRIDE



The amino acid positions and the hydrophobic layers (in bold) of the SNAP25 SNARE motif are numbered and indicated above the sequences.

VAMP2 (1-116): A DNA construct encoding GST-tagged mouse VAMP2 (pSK28) was described previously (Kedar et al., 2015). The GST-tag was removed by thrombin cleavage resulting in a short N-terminal extension highlighted in red.

GSMSATAATVPPAAPAGEGGPPAPPPNLTSNRRLQQTQAQVDEVVDIMRVNVDKVLERDQKLSELDDRADALQAGASQFETSAAKL KRKYWWKNLKMMIILGVICAIILIIIIVYFST

Synaptotagmin1: A DNA construct (pLM6) encoding His_6_-tagged rat Syt1 lacking the lumenal domain was described previously (Mahal et al., 2002). To replace the C-terminal His_6_-tag of this construct with a twin-Strep-tag (highlighted in red), the construct was subcloned into pPSG IBA103 (IBA Lifesciences) resulting in pSK151.

MGPWALIAIAIVAVLLVVTSAFSVIKKLLFKKKNKKKGKEKGGKNAINMKDVKDLGKTMKDQALKDDDAETGLTDGEEKEEPKEEEKLGKLQYSLDYDFQNNQLLVGIIQAAELPALDMGGTSDPYVKVFLLPDKKKKFETKVHRKTLNPVFNEQFTFKVPYSELGGKTLVMAVYDFDRFSKHDIIGEFKVPMNTVDFGHVTEEWRDLQSAEKEEQEKLGDICFSLRYVPTAGKLTVVILEAKNLKKMDVGGLSDPYVKIHLMQNGKRLKKKKTTIKKNTLNPYYNESFSFEVPFEQIQKVQVVVTVLDYDKIGKNDAIGKVFVGYNSTGAELRHWSDMLANPRRPIAQWHTLQVEEEVDAMLAVKKGSAWSHPQFEKGGGSGGGSGGSAWSHPQFEK

Complexin II wt: A DNA construct (pMDL80) encoding His_6_-tagged human CpxII was described previously (Malsam et al., 2012). The His_6_-tag was removed by thrombin cleavage (short N-terminal extension highlighted in red).

GSHMDFVMKQALGGATKDMGKMLGGEEEKDPDAQKKEEERQEALRQQEEERKAKHARMEAEREKVRQQIRDKYGLKKKEEKEAEEKAALEQPCEGSLTRPKKAIPAGCGDEEEEEEESILDTVLKYLPGPLQDMFKK

Complexin II BPA mutants: For site-directed insertion of the unnatural amino acid p-benzoyl-phenylalanine (BPA), the corresponding codons of the CpxII sequence were replaced with the amber stop codon (TAG) using pMDL80 as template DNA.

Complexin II VAMP2- and SNAP25 binding mutants: pMDL80 was used as template DNA to generate the VAMP2- (K33E, R37E, A40K, Q44E, resulting in pSK130) and the SNAP25 (D27R, A30R, Q31E, E34R)-binding mutants.

Complexin II fragments for circular dichroism measurements: the residues 21-83 of the Cpx II wt sequence and the corresponding CpxII-VAMP2- and SNAP25 binding mutants were subcloned into pCA528 resulting in pJM112, pJM113 and pJM114, respectively.

#### Protein expression and purification

All proteins were expressed in *E.coli* BL21(DE3). Cell disruption was performed with the high pressure pneumatic processor 110L (Microfluidics). The concentration of purified proteins was determined using SDS-PAGE and Coomassie blue-staining employing BSA as standard protein and ImageJ software for quantification. Light chains of botulinum neurotoxins B and D expression vectors were kind gifts of Dr. Thomas Binz and late Dr. Heiner Niemann. t-SNARE expression and purification was performed as described previously (Parlati et al., 1999). VAMP2 was expressed and purified as described previously (Kedar et al., 2015). Synaptotagmin1 containing a C-terminal twin-strep-tag was expressed and purified as described previously for the His_6_-tagged protein (pLM6) (Malsam et al., 2012) with the following modifications: imidazole was omitted from all buffers and 50 mM biotin was used for protein elution from Strep-Tactin XT superflow high capacity resin. Complexin II wt and the Complexin II SNARE-binding mutants were expressed and purified as described previously (Malsam et al., 2012). Complexin II fragments containing an N-terminal His_6_-SUMO tag were expressed and bound to Ni-NTA agarose as described for Complexin II wt. The His_6_-SUMO tag was removed by SUMO protease cleavage (Thermo Fisher Scientific cat.no 12588018) at 4°C for 16 hours following the manufacturer’s instructions.

Complexin II BPA mutants were expressed using the pEVOL/pET system (Young et al., 2010). *E*. *coli* BL21(DE3) was co-transformed with the pEVOL-pBpF plasmid and the pET15b expression plasmid carrying the respective CpxII amber mutant. 1 l LB media supplemented with a 50 mM potassium phosphate buffer (pH 7.3) was inoculated to grow a bacterial log-phase culture to an OD of 0.6. The culture was then transferred into a 2 l beaker glass with a magnetic stir bar to rapidly chill to 30°C using an ice-water-bath and supplemented with 1 mM BPA by the addition of 10 mL 100× BPA stock solution (100 mM BPA dissolved in 100 mM NaOH). The culture was then transferred back into a 5 l conical shake flask and incubated at 30°C. At an OD_600_ of 1.0 arabinose was added to a final concentration of 30 mM to induce tRNA transcription and expression of BPA-aminoacyl-tRNA synthetase for 1 hour at 30°C, followed by the induction of complexin expression with 0.3 mM IPTG for another 4 hours at 30°C. Purification of all BPA-containing complexin mutants was performed as described for Complexin II wt.

#### Preparation of small unilamellar v-SNARE/Synaptotagmin1 vesicles

Fluorophore-labeled lipids (Atto488-DPPE and Atto550-DPPE) were obtained from ATTO-TEC. All other lipids were from Avanti Polar Lipids. VAMP2/Syt1 lipid mix (3 μmol total lipid): 15 mol% DOPS (1,2-dioleoyl-sn-glycero-3-phosphoserine), 28.5 mol% POPC (1-palmitoyl-2-oleoyl-sn-glycero-3-phosphocholine), 25 mol% POPE (1-hexadecanoyl-2-octadecenoyl-sn-glycero-3-phosphoethanolamine), 5 mol% liver PI (L-α-phosphatidylinositol), 25 mol% cholesterol (from ovine wool), 0.5 mol% Atto488 1,2-dipalmitoyl-sn-glycero-3-phosphoethanolamine, 0.5 mol% Atto550 1,2-dipalmitoyl-sn-glycero-3-phosphoethanolamine.

VAMP2/Syt1 SUVs were formed in the presence of VAMP2 (protein-to-lipid ratio 1:350) and Syt1 (1:700) using the lipid mix defined above and the previously described technique of dilution and dialysis followed by a Nycodenz gradient centrifugation (Weber et al., 1998). 3 μmol dried VAMP2/Syt1 lipid mix were dissolved with VAMP2 in reconstitution buffer (20 mM HEPES-KOH, pH 7.4, 400 mM KCl, 1.7% octyl-b-D-glucopyranoside, 1 mM DTT) in a final volume of 0.7 ml at 30°C for 30 minutes. While vortexing vigorously, 0.3 mL Syt1 was added, followed by the rapid addition of 2 mL detergent-free reconstitution buffer to form SUVs. Detergent was removed by over night dialysis against 5 l of fusion buffer (20 mM HEPES-KOH, pH 7.4, 135 mM KCl, 1 mM DTT) at 4°C using Spectrapore 6–8 kDa cutoff dialysis tubing. The SUVs were further purified and concentrated by flotation in a Nycodenz (PROGEN) step gradient. 3 mL dialysate was mixed with 3 mL of 80% (w/v) Nycodenz dissolved in fusion buffer and then divided equally into two 11 × 60 mm ultraclear centrifuge tubes (Beckman). Then, each was overlaid with 750 μl 35% (w/v) Nycodenz, followed by 150 μL 12% Nycodenz and 100 μL fusion buffer lacking Nycodenz. After centrifugation in a SW60 rotor (Beckman) at 55,000 rpm for 4 hr at 4°C, the vesicles were harvested from the 0/12% Nycodenz interface in volumes of 250 mL per tube, pooled and dialyzed for another 18 hours against 5 l fusion buffer. The vesicles were snap-frozen in liquid nitrogen and stored at —80°C. Protein amounts in the reconstituted liposomes were determined by SDS–PAGE analysis and Coomassie blue staining using BSA protein standards followed by quantification with ImageJ 1.43u (National Institutes of Health) software. Lipid recoveries were determined by the quantification of Atto550 fluorescence.

#### Preparation of giant unilamellar t-SNARE vesicles

Fluorophore-labeled lipids (Atto647-DPPE) were obtained from ATTO-TEC. All other lipids were from Avanti Polar Lipids. Syntaxin1A/SNAP25 lipid mix (5 μmol total lipid): 15 mol% DOPS (1,2-dioleoyl-sn-glycero-3-phosphoserine), 35 mol% POPC (1-palmitoyl-2-oleoyl-sn-glycero-3-phosphocholine), 20 mol% POPE (1-hexadecanoyl-2-octadecenoyl-sn-glycero-3-phosphoethanolamine), 4 mol% liver PI (L-α-phosphatidylinositol), 1 mol% brain PI(4,5)P2 (L-α-phosphatidylinositol-4,5-bisphosphate), 25 mol% cholesterol (from ovine wool), 0.05 mol% Atto647 1,2-dipalmitoyl-sn-glycero-3-phosphoethanolamine. 5 μmol dried Syntaxin1A/SNAP25 lipid mix were dissolved with 5 μmol t-SNARE complex (protein-to-lipid ratio 1:1000) in a final volume of 0.7 ml containing 1.7% octyl-β-D-glucopyranoside at 30°C for 30 minutes. While vortexing vigorously, t-SNARE SUVs were formed by the rapid addition of 1.6 mL detergent-free reconstitution buffer (20 mM HEPES-KOH, pH 7.4, 400 mM KCl, 1 mM DTT). A PD-10 G-25 desalting column (GE Healthcare) was equilibrated with drying buffer (1 mM HEPES-KOH, pH 7.4, 20 mM Trehalose, 1% glycerol, 1 mM DTT) to remove excess salt and monomeric detergent applying the spin protocol. Desalted SUVs were snap frozen in four aliquots of 1.25 μmol each in liquid nitrogen and stored at —80°C. One aliquot t-SNARE SUVs was rapidly thawed at 37°C and desalted using a PD MidiTrap G-25 desalting column (GE Healthcare) equilibrated with drying buffer to remove trace contaminants of detergent using the gravity protocol. 1.4 ml eluate were collected and liposomes sedimented in a SW-55 rotor (Beckman) using 11×39 mm polyallomer tubes (Beckman) with adaptors (Beckman) at 35.000 rpm for 2 h at 4°C. The liposome pellet was resuspended in a total volume of 20 μl and spread as uniform layer (15 mm diameter) on the surface of Pt-coated glass slides (GeSIM). The liposome suspension was dried for 1 h at low vacuum (100 mbar). A PTFE O-ring (18 × 2 mm) (Dichtelemente arcus GmbH) was used to seal the chamber and electroformation of GUVs was performed in GUV preparation buffer (0.5 mm EPPS-KOH [3-[4-(2-hydroxyethyl)-1-pi-perazinyl]propanesulfonic acid hydrate], pH 8.0, 0.25 M sucrose (Ca^2+^ free from FLUKA), 1 mM DTT) for 15 h at 10 Hz and 1 V at 0°C. Around 500 μl GUV suspension were removed from the glass plate by gentle pipetting and used in subsequent assays. The GUVs could be stored at 0°C without apparent loss of activity for up to 7 days. Lipid recoveries were determined by the quantification of Atto647 fluorescence.

#### Fusion assays

Fusion buffer was 20 mM MOPS-KOH, pH 7.4, 135 mM KCl, 1 mM DTT (dithiothreitol). In all cases, t-SNARE-GUVs (125 μM lipid) and v-SNARE/Syt1-SUVs (25 μM lipid) were used in a final volume of 100 μl; liposomes were mixed with 100 μM EGTA-KOH, pH 7.4 and 0.5 μM MgCl_2_ in the presence or absence of CpxII and pre-incubated for 5 minutes on ice before quickly transferring to a prewarmed 96 well microplate (Sigma-Aldrich). Samples were measured at 37°C in a Synergy 4 plate reader (BioTek Instruments GmbH) at intervals of 10 s. After 2 min, Ca^2+^ was added to a final concentration of 100 μM by the addition of 20 μl of a 6 × stock solution (6.6 mM Ca^2+^, 5.5 mM EGTA) and the measurement continued for another 5 minutes. Free Ca^2+^ concentrations were calculated by using the Ca-EGTA Calculator v1.3 software (Maxchelator) (Schoenmakers et al., 1992). After recording the fusion kinetics, the reactions were mixed with detergent (final concentrations 1% SDS/0.4% dodecylmaltoside (Fluka) and incubated for another 5 minutes at 37°C to determine the maximal fluorescent signals. The Atto488 fluorescence values of a fusion kinetics were normalized to the maximal fluorescent signal of the individual measurement set. Normalized fluorescent values obtained from control incubations containing v-SNARE/Syt1-SUVs pretreated with BoNT/D were subtracted from individual measurement sets. Fusion kinetics displayed in all figures show the mean of three experiments. Changes in fusion kinetics in response to the Ca^2+^ trigger were quantified as change in % of total fluorescence 10 sec after the addition of Ca^2+^.

#### Liposome cross-linking

All solutions were adjusted to 280 mOsm. t-SNARE GUVs (250 μM lipid) were mixed with VAMP2-Syt1 SUVs (50 μM lipid) and 6 μM CpxII wt or BPA mutants in fusion buffer (20 mM MOPS-KOH, pH 7.4, 135 mM KCl, 0.5 mM MgCl_2_, 100 μM EGTA-KOH, 5 mM glutathione, 1 mM DTT) in a final volume of 100 μl and incubated for 30 minutes on ice. To remove unbound SUVs and complexin, the reaction-mix was overlayed on 100 μl of 70 mM sucrose in fusion buffer and underlayed with a cushion of 5 μl 250 mM sucrose in 1 mM HEPES-KOH, pH 7.4. GUVs were re-isolated by centrifugation at 10.000 × g for 15 minutes in an A-8-11 swing-bucket rotor (Eppendorf). The supernatant was removed and sedimented liposomes were gently resuspended in a total volume of 15 μl for subsequent cross-linking. Samples were irradiated on ice with a UV-LED lamp (Opsytec Dr. Gröbel GmbH) at 365 nm applying 15 pulses of 1 s at maximum irradiation power (25 W/cm^2^), inserting pauses of 2 s between pulses.

#### Protease treatment of liposomes with Botulinum neurotoxins

Liposome samples were prepared and Cpx R37BPA arrested SUV-GUV complexes re-isolated as described above for liposome cross-linking. 15 μl sedimented liposomes were diluted with 20 μl fusion buffer and heated at 98°C for 1 minute, followed by the addition of 10 μl 2% (w/v) Triton X-100. Solubilized proteins were subjected to site-specific proteolytic cleavage of VAMP2 with 0.5 μM BoNT/B or BoNT/D in final volumes of 50 μl at 37°C for 15 minutes. Samples were then mixed with 500 μl cold acetone and incubated for 3 hours at —20°C. Precipitated proteins were sedimented at 0°C for 30 minutes at 20.000 × g. Pellets were air-dried, solubilized in SDS-PAGE sample buffer and proteins separated on 16% Tris-Tricine gels for Coomassie brilliant blue staining or Western-blot analysis.

#### Circular dichroism spectroscopy

Purified CpxII protein fragments (residues 21-83) were diluted with PBS to a final concentration of 50 μM. The circular dichroism spectra were recorded on a Jasco J715 spectropolarimeter at 25°C. A cell with a 1-mm path length was used for spectra recorded between 190 and 250 nm with sampling points every 0.2 nm. For each measurement, the average of five spectra was taken. Units are expressed in mean molar ellipticity per residue.

#### Cryo-electron microscopy and cryo-tomography

Samples were processed for plunge freezing as described in Malsam et al. (2012). Samples were imaged at cryo temperatures under standard low-dose conditions on either a FEI Tecnai Spirit electron microscope (120 kV, 23000×) equipped with a Gatan Ultrascan 4000 CCD camera (4.9 Å /pixel, Gatan, Pleasanton CA) or on a FEI Tecnai Polara electron microscope (300 kV, 23000x) equipped with a Falcon 2 direct electron detection camera (4.85 Å /pixel). Imaging of each sample was performed in an unbiased, semiautomated manner using SerialEM (Bharat et al., 2014; Mastronarde, 2005). The number of SUVs bound to one GUV was recorded for each sample.

For morphological analysis of prefusion sites, cryo-tomograms were automatically collected on a FEI Titan Krios electron microscope operated at 300 kV and equipped with a Gatan Quantum 967 LS energy filter and a K2 ×p direct electron detector. Dose-symmetric tilt series (Hagen et al., 2017) were acquired in EFTEM mode with a 20eV slit and a calibrated magnification of 130kx. A tilt range of ± 66 with 3° angular increments was chosen, resulting in a total dose of approximately 130 electrons/Å^2^. Super-resolution frames were aligned on-the-fly with a frame-alignment algorithm built into SerialEM (Mastronarde, 2005) and Fourier cropped to 4k × 4k images giving a pixel size of 1.05 Å /pixel. Subsequently, tilt series were sorted and filtered by cumulative electron dose (Grant and Grigorieff, 2015; Schur et al., 2016). Tomograms were generated by weighted-back projection using the IMOD/etomo pipeline (Kremer et al., 1996). For visual inspection, tomograms were binned twice and low-pass filtered resulting in a final pixel size of 4.2 Å /pixel. Medium magnification grid square maps (6500×, 2.27 nm/pixel) generated during the imaging sessions were also used for docking analysis.

#### Cpx-SNARE cross-link products in SDS-sensitive and resistant SNARE complexes

Liposome reaction mixes were prepared and re-isolated for cross-linking as described above. Before or after cross-linking, 2 mM free calcium was added to trigger liposome fusion in a total sample volume of 20 μl. After the addition of SDS-PAGE loading buffer, the samples were heated to either 70°C or 100°C for 5 minutes before separating proteins by SDS-PAGE on 12% polyacrylamide gels.

#### Preparation of cultured hippocampal neurons

Murine microisland cultures were prepared as described (Xue et al., 2007). Cplx1-3 triple KO neurons were described previously (Xue et al., 2008). Animals were handled according to the rules of Berlin authorities and the animal welfare committee of the Charité – Universitätsmedizin Berlin, Germany. Primary hippocampal neurons were prepared from mice on embryonic day E18 and plated at 300 cm^-2^ density on WT astrocyte microisland for autaptic neuron electrophysiology. For western blotting hippocampal neurons were plated at 10.000 cm^-2^ on continental WT astrocyte feeder layer.

#### Lentiviral constructs and virus production

For expression of CpxII variants within neuronal cells, a modified lentiviral vector (Lois et al., 2002) was used containing a human Synapsin-1 promoter, driving the expression of a nuclear GFP and the CpxII variant (CpxII wt, VAMP2- or SNAP25-binding mutants of CpxII). The cDNAs were coupled via a self cleaving P2A site (Kim et al., 2011) leading to bicistronic expression of the 2 proteins (f(syn)NLS-GFP-P2A-CpxII-WPRE). Lentiviral particles were prepared by the Charité Viral Core Facility as previously described (Lois et al., 2002), v*cf.*charite.de. Briefly, HEK293T cells were cotransfected with the shuttle vector f(syn)NLS-GFP-P2A-CpxII-WPRE and helper plasmids, pCMVdR8.9 and pVSV.G with polyethylenimine. Virus containing supernatant was collected after 72 h, filtered, aliquoted, flash-frozen with liquid nitrogen, and stored at —80°C. For infection, about 5×10^5^-1×10^6^ infectious virus units were pipetted onto 1 DIV hippocampal Cplx1-3 triple KO neurons per 35 mm-diameter well.

#### Electrophysiology of cultured neurons

Whole cell patch-clamp recordings in autaptic glutamatergic neurons were performed as previously described (Trimbuch et al., 2014). The extracellular solution contained 140 mM NaCl, 2.4 mM KCl, 10 mM HEPES, 2 mM CaCl_2_, 4 MgCl_2_, 10 mM Glucose (pH adjusted to 7.3 with NaOH, 300 mOsm). The patch pipette solution contained 136 mM KCl, 17.8 mM HEPES, 1 mM EGTA, 0.6 mM MgCl_2_, 4 mM ATP-Mg, 0.3 mM GTP-Na, 12 mM phosphocreatine and 50 units/ml phosphocreatine kinase (300 mOsm, pH 7.4). Neurons were clamped at —70 mV with a Multiclamp 700B amplifier (Molecular Devices) under control of Clampex 9 (Molecular Devices) at DIV 14-17. Data were analyzed offline using Axograph X (AxoGraph Scientific) and Prism 7 (GraphPad Software). Statistic significances were determined by one-way analysis of variance (ANOVA) with Kruskal-Wallis test followed by Dunn’s post test to compare all groups.

EPSCs were evoked by a brief 2 ms somatic depolarization to 0 mV. EPSC amplitude was determined as the average of 6 EPSCs at 0.1 Hz. RRP size was determined by measuring the charge transfer of the transient synaptic current induced by a pulsed 5 s application of hypertonic solution (500 mM sucrose in extracellular solution). Pvr was calculated as the ratio of the charge from an evoked EPSC and the RRP size of the same neuron. Evoking 5 or 50 synaptic responses at 50 or 10 Hz respectively in standard external solution analyzed short-term plasticity. For analyzing mEPSCs, traces were digitally filtered at 1 kHz offline. Then the last 9 s of 5 traces of EPSCs at 0.1 Hz were analyzed using the template-based mEPSC detection algorithm implemented in Axograph X (AxoGraph Scientific) and substracted from background noise by detecting events in the last 4 s of 5 EPSCs at 0.2 Hz in 3 μM NBQX (Tocris) in extracellular solution.

### Quantification and Statistical Analysis

The ImageJ software (National Institutes of Health) was used for the quantification of protein amounts. To determine average values and standard deviations, the Excel software (Microsoft) was used. The number of experimental replicates and other information relevant for assessing the accuracy and precision of the analysis are presented in the accompanying legend of each figure.

## Supplementary Material

Supplemental Information can be found online at https://doi.org/10.1016/j.celrep.2020.107926.

Supp Info

## Highlights

- Detection of a bipartite complexin accessory helix-SNARE interface prior to fusion
- The Cpx-SNARE interface suppresses SNARE complex zippering
- The interface contributes to clamp spontaneous neurotransmitter release
